# KLF7/VPS35 axis contributes to hepatocellular carcinoma progression through CCDC85C-activated β-catenin pathway

**DOI:** 10.1186/s13578-021-00585-6

**Published:** 2021-04-15

**Authors:** Yarong Guo, Bao Chai, Junmei Jia, Mudan Yang, Yanjun Li, Rui Zhang, Shunmin Wang, Jun Xu

**Affiliations:** 1 Department of Oncology, The First Affiliated Hospital of Shanxi Medical University, Taiyuan, 030001 Shanxi China; 2 Department of Gastroenterology, Shanxi Academy of Medical Science, Shanxi Bethune Hospital, Taiyuan, 030032 Shanxi China; 3 Shanxi Provincial Cancer Hospital, Affiliated Cancer Hospital of Shanxi Medical University, Taiyuan, 030013 Shanxi China; 4 Department of Surgery, Shanxi Academy of Medical Science, Shanxi Bethune Hospital, Taiyuan, 030032 Shanxi China; 5 Department of Surgery, The First Affiliated Hospital of Shanxi Medical University, 85 South Jiefang Road, Taiyuan, 030001 Shanxi China

**Keywords:** Hepatocellular carcinoma, KLF7, VPS35, Ccdc85c, Β-catenin, LGK974

## Abstract

**Objective:**

Dysregulation of KLF7 participates in the development of various cancers, but it is unclear whether there is a link between HCC and aberrant expression of KLF7. The aim of this study was to investigate the role of KLF7 in proliferation and migration of hepatocellular carcinoma (HCC) cells.

**Methods:**

CCK8, colony growth, transwell, cell cycle analysis and apoptosis detection were performed to explore the effect of KLF7, VPS35 and Ccdc85c on cell function in vitro. Xenografted tumor growth was used to assess in vivo role of KLF7. Chip-qPCR and luciferase reporter assays were applied to check whether KLF7 regulated VPS35 at transcriptional manner. Co-IP assay was performed to detect the interaction between VPS35 and Ccdc85c. Immunohistochemical staining and qRT-PCR analysis were performed in human HCC sampels to study the clinical significance of KLF7, VPS35 and β-catenin.

**Results:**

Firstly, KLF7 was highly expressed in human HCC samples and correlated with patients’ differentiation and metastasis status. KLF7 overexpression contributed to cell proliferation and invasion of HCC cells in vitro and in vivo. KLF7 transcriptional activation of VPS35 was necessary for HCC tumor growth and metastasis. Further, co-IP studies revealed that VPS35 could interact with Ccdc85c in HCC cells. Rescue assay confirmed that overexpression of VPS35 and knockdown of Ccdc85c abolished the VPS35-medicated promotion effect on cell proliferation and invasion. Finally, KLF7/VPS35 axis regulated Ccdc85c, which involved in activation of β-catenin signaling pathway, confirmed using β-catenin inhibitor, GK974. Functional studies suggested that downregulation of Ccdc85c partly reversed the capacity of cell proliferation and invasion in HCC cells, which was regulated by VPS35 upregulation. Lastly, there was a positive correlation among KLF7, VPS35 and active-β-catenin in human HCC patients.

**Conclusion:**

We demonstrated that KLF7/VPS35 axis promoted HCC cell progression by activating Ccdc85c-medicated β-catenin pathway. Targeting this signal axis might be a potential treatment strategy for HCC.

## Introduction

Hepatocellular carcinoma ranks as the most frequently occurring type of malignancy with poor survival and high mortality, which constitutes 7% of all the cancers worldwide [1, 2]. HCC typically emerges on the setting of chronic liver disease at a rate dependent upon the complex interplay between the disease, genetic and environmental factors. Rapid advance in medical and surgical technologies has continuously resulted in the improving treatment results [3]. Unfortunately, under current management, patients with advanced HCC have limited treatment options and substantially poor progress [4]. Thus, further explicating into the sophisticated pathogenesis of HCC might lead to the continued evolution of more effective therapeutic strategies.

Krüppel-like factors (KLFs) are a class of high conserved zinc finger containing transcription factors, which are extensively involved in important biological processes of human beings [5]. KLFs play important roles in regulating gene expression via binding to the GC rich elements of targeted gene promoters [6]. Dysregulation of KLF family proteins participates in the development of various cancers. For example, KLF7 serves as an independent predictor of poor prognosis in pediatric acute lymphoblastic leukemia, where enhanced expression of KLF7 inhibited hematopoietic stem and progenitor cell function [7]. KLF5 contributes to cell migration of bladder cancer cells by up-regulating FYN expression [8]. KLF7 functions as a crucial regulator in cellular growth and differentiation in multiple types of organs [9]. Actually, There are growing evidence revealing that KLF7 is aberrantly expressed in a variety of human solid tumors and plays pivotal roles in proliferation, migration, invasion and EMT, poor prognosis of cancer cell lines [10, 11]. However, it is unclear whether there is a link between HCC and aberrant expression of KLF7.

Vacuolar protein-sorting-associated protein 35 (VPS35) is a component of the retromer complex and medicates the endosome-to-plasma membrane sorting and recycling of transmembrane receptor [12]. Mutation of VPS35 has emerged as a novel cause of autosomal dominant Parkinson's disease (PD), identified by exome sequencing [13]. Recently, it has been demonstrated that VPS35 participates in hepatocellular carcinoma tumor growth by activating PI3K/AKT signaling pathway [14]. Moreover, somatic copy number variations (CNV) spanning VPS35 was identified in several tumors [15]. Nevertheless, the precise mechanism of VPS35 as an oncogene in HCC development and progression need fully investigated.

Coiled-coil domain containing 85c (Ccdc85c) belongs to a family which consists of Ccdc85a, Ccdc85b and Ccdc85b. Ccdc85c contains a pair of conserved coiled-coli motif, which can regulate DNA transcription [16]. Of interest, mutation of Ccdc85c is correlated to hydrocephalus with frequent brain hemorrhage [17]. Another previous study revealed that the expression of Ccdc85c might be associated with cell proliferation of simple epithelial cells [17]. Until now, there was no study mentioned the functions of Ccdc85c in HCC development.

Here, we identified that KLF7 was overexpressed in HCC. Using “gain of function” and “loss of function” strategies in vitro and in vivo, we revealed that KLF7 promoted tumor growth and invasion in HCC cell lines. Furthermore, CHIP-qPCR determined that KLF7 bound to the promoter of Ccdc85c to regulate its expression. Thus, our findings confirmed that KLF7 aggravated HCC progression through the VPS35/Ccdc85c/β-catenin axis.

## Materials and methods

### Patients

HCC tissues were collected from the patients before any intervention. Informed consent was obtained from each patient. This study was approved by the Human Ethics Committee of The First Affiliated Hospital of Shanxi Medical University. The experiments were conducted according to the protocols of the World Health Organization (WHO) criteria.

### Immunohistochemical (IHC) staining of HCC tissue microarray

IHC staining of KLF7, VPS35 and active-β-catenin was performed in human HCC tissues microarray (containing 50 cancer and 20 normal tissues), according to the protocol as described previously [18].

### Cell culture

The human HCC cell lines Huh-7 and SKHEP1 were originally obtained from the ATCC. The cells were cultured in DMEM and RPMI-1640 medium (Hyclone), added with 10% fetal bovine serum (FBS, Gibco), 100 U/ml penicillin and 100 mg/ml streptomycin (Gibco). All cells were maintained in a 37 ℃, 5% CO_2_ incubator.

### Cell proliferation assays

Cell proliferation reagent kit (CCK-8) was applied for determining cell proliferation capacity of HCC cell lines. Transfected cells were seeded in 96-well plates with a density of 1500 cells/well. Then, cell viability was assessed every 24 h and compared between different groups, according to the manufacturer’s introductions.

### Colony formation assay

To assess the capacity of colony formation, transfected cells were plated in each well of 6-well plates with a density of 1000 cells, maintained at 37 ℃ and the cell culture medium was changed every 3 days. After incubation for 10 days, the colonies were fixed and stained with 0.1% crystal violet solution. The colonies containing at least 50 cells were considered viable and then analyzed from different groups of three independent wells.

### Transwell assay

Transwell assay was performed to assess the migration capacity of HCC cell lines under different treatments. Briefly, 1.0 × 10^5^ cells in 200 μl serum-free medium were plated into the upper chamber with a Matrigel-coated membrane (Corning lnc., USA), while 600 μl medium supplemented with 10% FBS was loaded into the lower chamber. After 24 h of incubation at 37 ℃, the migrated cells were fixed with 20% methanol and stained with 0.1% crystal violet solution. Then the cells were visualized and imaged under a microscope in at least 5 random fields (Olympus, Japan).

### Cell apoptosis assay

The transfected cells in early and late apoptosis stages were quantified with double stained FITC-Annexin V and PI, using FITC Annexin V Apoptosis Detection Kit (BD Biosciences). Briefly, cells were collected and resuspended with 500 μl binding buffer, following by incubated with Annexin V and PI in dark situation for 15 min. Then the stained cells were analyzed using BD FACS flow cytometer (BD Biosciences). The percentage of apoptotic cells were calculated and compared between different groups.

### Cell cycle assay

Flow cytometric analysis was applied to examine DNA content after different treatments. The transfected cells were harvested and fixed with 75% pre-cold ethanol at −20 ℃ overnight. The fixed cells were washed with PBS for three times, incubated with ribonuclease A in a 37℃ incubator for 30 min. After that, the cells were stained with PI for 30 min at room temperature and cell cycle profiles were determined by flow cytometer. The experiment was repeated independently at least three times.

### In vivo* study*

6–8 weeks, male BALB/c mice were obtained from Beijing Weitonglihua company (Beijing, China) and housed in a SPF environment. 5 × 10^6^ MHCC97H cells stably transfected with shRNA-KLF7 and shRNA-Ctrl, and 2 × 10^6^ MHCC97H cells stably transfected with pcDNA3.1-KLF7 vectors or negative control were subcutaneously injected into the right collar of the nude mice, respectively. Each group contains of 5 mice and tumor size was measured every 3 days. After 30 days, tumor volume was calculated according to the formula: V = ab^2^/2, where a states longer tumor diameter and b states shorted tumor diameter.

### Co-IP assay

Transfected cells were lysed with RIPA lysis buffer and prepared for Co-IP assay. After the protein A/G beads incubated with the primary antibody or IgG at 4 ℃ for 6–8 h, the prepared cell lysate was added to the mixture of beads and antibody overnight at 4 ℃. Then, the above mixture washed with PBS containing 0.1% NP40 for three times and eluted using the elution buffer. Finally, the elution samples were detected by SDS-PAGE, companying with silver stain (Beyotime, china) and mass spectrometry analysis.

### ChIP-qPCR

ChIP-qPCR assay was used to assess whether KLF7 interacts with the promoter sequence of VPS35. Briefly, pCDNA3.1 containing KLF7 CDS with a flag sequence was transfected into HCC cells. ChIP-qPCR experiments were performed by using SimpleChIP enzymatic chromatin IP kit (Cell Signaling), following the indicated protocols. The primer sequences of qPCR were as follow: forward, 5′-TGCTTTTCCGAGTTTTGTTTGTT-3′ and reverse, 5′-GACCTTAACCGGGCAATGC-3’.

### Luciferase reporter assay

For binding assessment of transcription factor KLF7 to the target gene promoter, the VPS35 promoter sequence was amplified by PCR and cloned into luciferase reporter vector. Then the luciferase reporter vector and KLF7 full-length vector were co-transfected into HCC cells using Lipo2000 (Invitrogen, USA). The relative luciferase activity was examined by a dual luciferase reporter assay system (Promega, USA).

### Western blot assay

For western blot assay, HCC cell lines after transfection or without processing were harvested in lysis buffer. The concentration of total protein was evaluated by BCA protein assay kit (Thermo Fisher Scientific, lnc.). Then the protein was separated by 12% SDS-PAGE and transferred onto PVDF membranes (Thermo Fisher Scientific, lnc.). After incubation with 5% milk at room temperature for 2 h, the membranes were maintained with primary antibody as indicated at 4 ℃ overnight and then incubated with secondary antibodies at room temperature for 2 h. Protein bands were visualized with an enhanced chemiluminescence (ECL) assay. (Millipore, Billerica, MA). GAPDH was acted as endogenous control. The primary antibodies used as follow: anti-KLF7 (398576, Santa Cruz), anti-GAPDH (60004–1-Ig, Proteintech), anti-VPS35 (157220, Abcam), anti-CCDC85C (TA330857, ORIGENE), anti-active β-catenin (19807, Cell Signaling Technology), anti-β-actin (47778, Santa Cruz).

### qRT-PCR assay

qRT-PCR experiment was carried out according to the protocols as described previously [18]. qPCR primer sequences were as follow: KLF7 forward, 5′-AGACATGCCTTGAATTGGAACG-3′ and reverse, 5′-GGGGTCTAAGCGACGGAAG-3′. GAPDH forward, 5′-TGACTTCAACAGCGACACCCA-3′, and reverse, 5′-CACCCTGTTGCTGTAGCCAAA-3’.

### Statistical analyses

Statistical analyses were performed using SPSS statistics version 22.0 and GraphPad Prism 8.0. Data are presented as the mean ± standard deviation. Generally, two-tailed student’s t-test or one-way ANOVA was performed to calculate significant differences between two groups or more than two groups, respectively. Each experiment was repeated independently at least three times. P < 0.05 was considered as statistically significant.

## Results

### KLF7 is overexpressed in human HCC patients

**Fig. 1:**
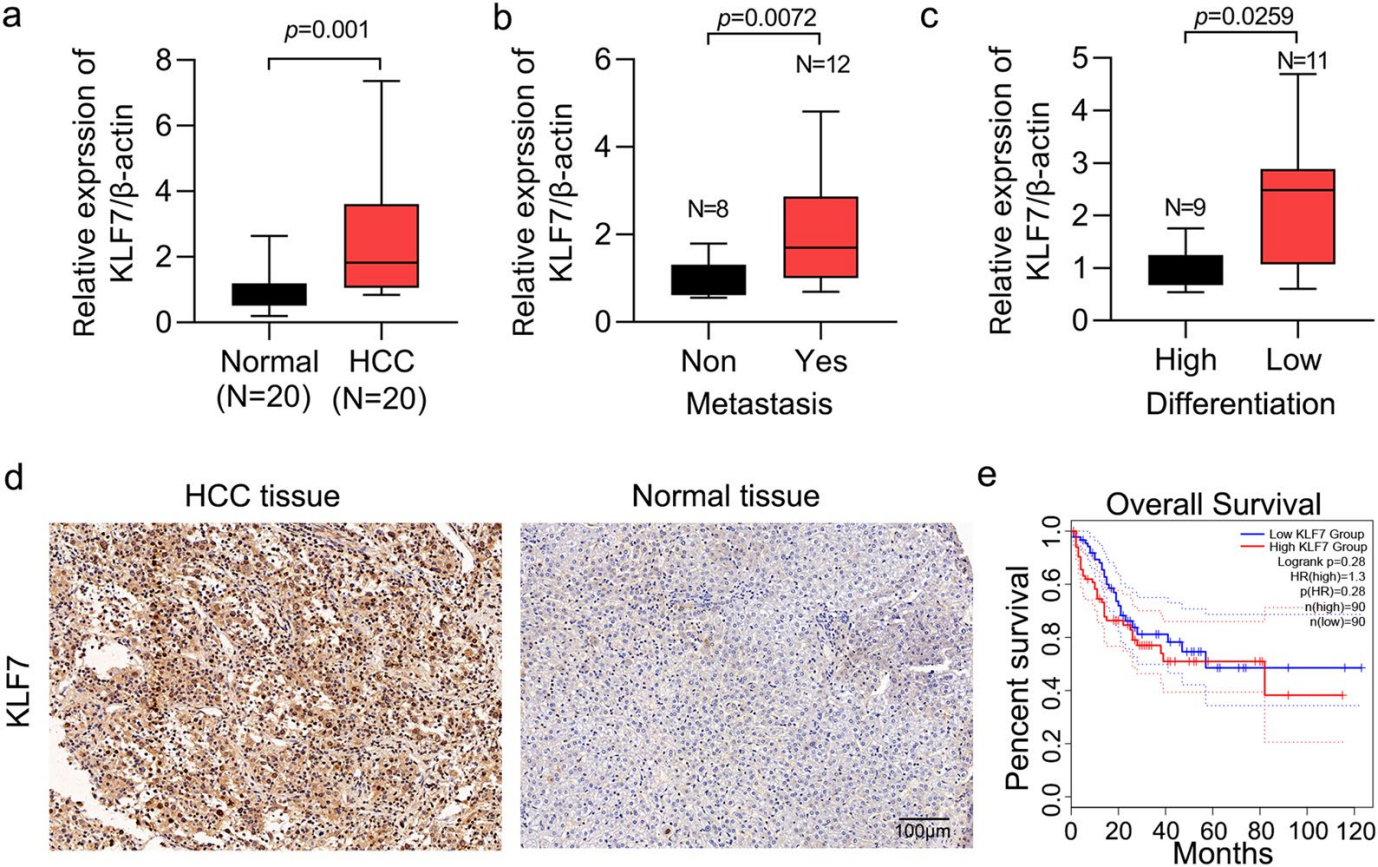
KLF7 was overexpressed in human HCC samples. **a** qRT-PCR assay examined KLF7 expression in 20 pairs of human HCC and adjacent normal samples. p = 0.001. **b** qRT-PCR assay examined KLF7 expression in HCC tissues with or without metastasis. p = 0.0072. **c** qRT-PCR assay examined KLF7 expression in HCC tissues of high and low differentiation status. p = 0.0259. **d** IHC staining examined KLF7 expression in HCC and normal samples. **e** Overall survival was analyzed in HCC patients who were divided into KLF7 high and low expression group (n = 90 per group). p = 0.28

Firstly, we explored the expression of KLF7 in human HCC patients. We collected cancer and adjacent normal tissues from 20 HCC patients. qRT-PCR results suggested that KLF7 was overexpressed in the cancer tissues (Fig. 1a). In addition, KLF7 expression was relatively higher in HCC patients with metastasis and low differentiation (Fig. 1b and c). Then, we performed IHC staining of KLF7 in HCC tissue microarray, which contained 50 cancer tissues and 20 normal tissues. We showed that KLF7 was strongly expressed in cancer tissues, in which the cell nucleus was stained with KLF7 antibody. By contrast, normal tissues showed low expression of KLF7 (Fig. 1d and Table 1). In addition, KLF7 high expression was associated with low differentiation status of the patients. The patients with metastasis had relatively higher KLF7 levels comparing with those without metastasis (Table 2). Because there was no survival information for these patients, we analyzed the association between KLF7 expression and patients’ survival from TCGA database. However, KLF7 high expression did not predict poor survival of the patients (Fig. 1e). Therefore, KLF7 is a predictor for HCC differentiation and metastasis, but not for patients’ survival.

**Table 1:** Protein level of KLF7, active β-catenin and VPS35 in HCC tissues and normal tissues

	Normal Tissue	Tumor tissue	χ^2^	p value
KLF7 High	6	30	5.15	0.023
KLF7 Low	14	20		
active β-catenin High	7	36	8.25	0.004
active β-catenin Low	13	14		
VPS35 High	8	33	3.98	0.046
VPS35 Low	12	17		
Total	20	50

**Table 2:** Correlation of KLF7 expression with clinicopathological characteristics in 50 patients of HCC

	Characteristic	KLF7 expression		p value
		High	Low	
Age	< 60	17	12	0.815
	≥ 60	13	8	
Gender	Male	18	10	0.485
	Female	12	10	
Differentiation	Well	9	13	0.015
	Poorly	21	7	
T classification	T1-T2	10	12	0.063
	T3-T4	20	8	
Lymph node metastasis	No	9	12	0.035
	Yes	21	8	
Tumor size	< 6	11	10	0.349
	≥ 6	19	10	

### *KLF7 silencing suppressed cell proliferation and invasion*, *and induced cell cycle arrest and apoptosis in HCC cell lines*

**Fig. 2:**
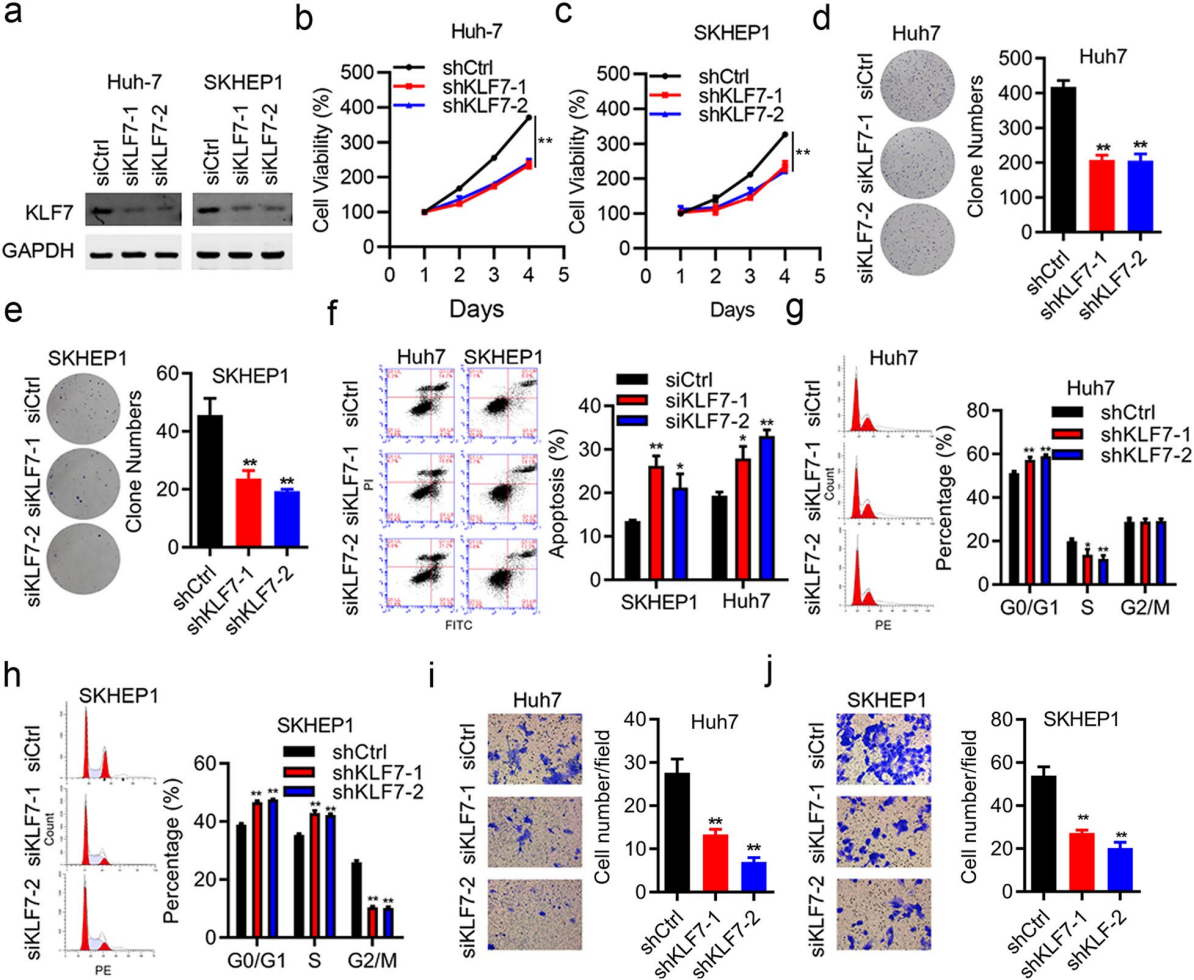
Downregulation of KLF7 inhibited HCC cell proliferation, invasion and induced cell cycle arrest and apoptosis. **a** Western blot assay examined knockdown of KLF7 expression mediated by siRNA in two HCC cell lines (Huh-7 and SKHEP1). **b**, **c** CCK-8 assay was performed to determine the proliferation capacity of KLF7-silencing Huh-7 (b) and SKHEP1 cells (c) after transfection with siRNA or siCtrl. **d**, **e** Colony formation assay was applied to measure the proliferation of si-KLF7-transfected Huh-7 and SKHEP1 cells. **f** Flow cytometry assay was used to detect cell apoptosis rate in Huh-7 and SKHEP1 cells. **g**, **h** Flow cytometry assay was conducted to identify the stages of cell cycle progression in Huh-7 and SKHEP1 cells. **i**, **j** Transwell assay was used to examine cell invasion in Huh-7 and SKHEP1 cells transfected with si-KLF7 and siCtrl. Each experiment was independently repeated at three times. *p < 0.05, **p < 0.01

To reveal the role of KLF7 in the proliferative capacity, we examined knockdown efficiency of KLF7 medicated by siRNA technology, in Huh-7 and SKHEP1 cell lines (Fig. 2a). After KLF7 knockdown, CCK-8 assay confirmed that knockdown of KLF7 obviously inhibited cell proliferation in both cell lines (Fig. 2b and c). Furthermore, the colony formation assay implied that the rates of colony formation of KLF7-silencing cells were much lower than those transfected with si-NC (Fig. 2d, e). These results suggested that KLF7 silencing repressed cell growth in HCC cells.

In addition, flow cytometry assay was performed to identify the stages of cell cycle progression in both HCC cell lines transfected with si-KLF7 or si-NC. The results indicated that Huh7 and SKHEP1 cells transfected with si-KLF7 both result in an obvious increase in the percentages of cells at G0/G1 phase, a marked decrease in S phase in Huh7 cells, while an increase in S phase and a remarkable decrease at G2/M phase in SEHEP1 cells (Fig. 2g, h, All p < 0.01). There was no obvious alteration of G2/M phase in Huh7 cells. These results indicated that knocking down KLF7 could lead to Go/G1 arrest. To clarify the potential molecular mechanism underlying the inhibition of cell growth after KLF7 knockdown, FITC-Annexin V and PI doubling staining assay was conducted to determine the effect of KLF7 downregulation on HCC cell apoptosis. As Fig. 1f revealed, the percentage of apoptosis cells in both Huh7 and SKHEP1 cells transfected with si-KLF7 were higher than those transfected with Huh-siNC (si-KLF7-1, p < 0.01; si-KLF7-2, p < 0.05) or SKHEP1-siNC (si-KLF7-1, p < 0.05; si-KLF7-2, p < 0.01). These data indicated that downregulation of KLF7 promote cell apoptosis in HCC cell lines, which revealed that KLF7 contributed to tumor progression through suppressing apoptosis.

Since metastasis process of cancer cell is recognized as an important indicator of tumor progression, we explored the capacity of cell invasion regarding transfection of siKLF7 or siNC into HCC cells. By Transwell assay in Huh-7 and SKHEP1 cell lines, cell invasion was clearly repressed upon KLF7 knockdown (Fig. 2i, j, all p < 0.01). Collectively, these data suggested that KLF7 could regulate cell process, including cell proliferation, invasion, cell cycle and cell apoptosis of HCC cell lines.

### Overexpression of KLF7 aggravates HCC cell progression

**Fig. 3:**
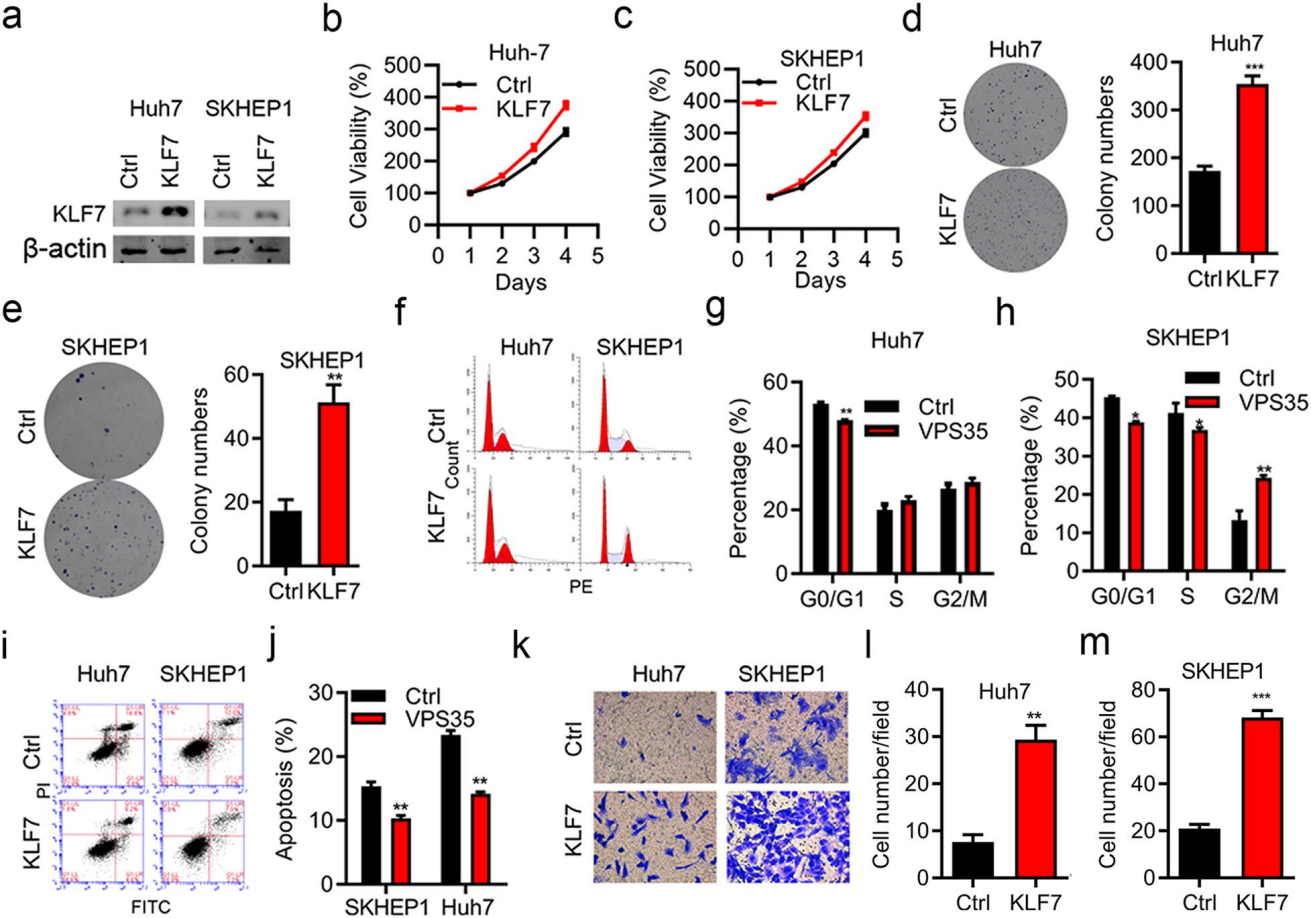
Overexpression of KLF7 aggravates cell progression in HCC cell lines. **a** Overexpression efficiency of KLF7 in Huh-7 and SHEP1 cells was confirmed by western blot. **b**, **c** Cell viability of KLF7-overexpressing Huh-7 and SHEP1 cells was determined by CCK-8 assay. **d**, **e** Colony formation of KLF7-overexpressing Huh-7 (p < 0.001) and SHEP1 (p < 0.01) cells were detected by colony formation assay. **f** Flow cytometry was preformed to detect the progression stages of cell cycle in KLF7-overexpressing Huh-7 and SHEP1 cells. **g**, **h** Statistical results of cell cycle in Huh-7 (p < 0.01) and SHEP1 cells (p < 0.01). **i**, **j** Cell apoptosis was determined after the transfection of pcDNA3.1-KLF7 or empty vector into Huh-7 and SHEP1 cells. **k** Representative images of transwell assay. **l**, **m** Transwell invasion assay of Huh-7 (p < 0.01) and SHEP1 (p < 0.001) cells transfected with KLF7-overexpressing vectors or empty vectors. *p < 0.05, **p < 0.01, ***P < 0.001

Correspondingly, to further unravel the oncogene roles of KLF7 in HCC development, KLF7 was remarkably upregulated in Huh-7 and SKHEP1 cells by pcDNA3.1-KLF7 plasmid transfection (Fig. 3a). CCK-8 assay and colony formation assay results showed that KLF7 overexpression enhanced cell proliferation as well as colony formation ability in both cell lines (Fig. 3b–e). In addition, flow cytometry assay in Huh-7 and SKHEP1 cells showed that overexpressing of KLF7 effectively promoted cell cycle progression (Fig. 3f–h), while inhibited cell apoptosis (Fig. 3i, j, all p < 0.01). Cell migration was also increased in Huh-7 and SKHEP1 cell lines, transfected with KLF7 overexpressing vectors (Fig. 3k-m, p < 0.01 for Huh-7 cells, p < 0.001 for SKHEP1 cells). These results indicated that KLF7 overexpression obviously promoted cell proliferation, invasion, and contributed to cell cycle and suppressed cell apoptosis of HCC cell lines.

### *KLF7 expression affects the growth of HCC transplanted tumors *in vivo

**Fig. 4:**
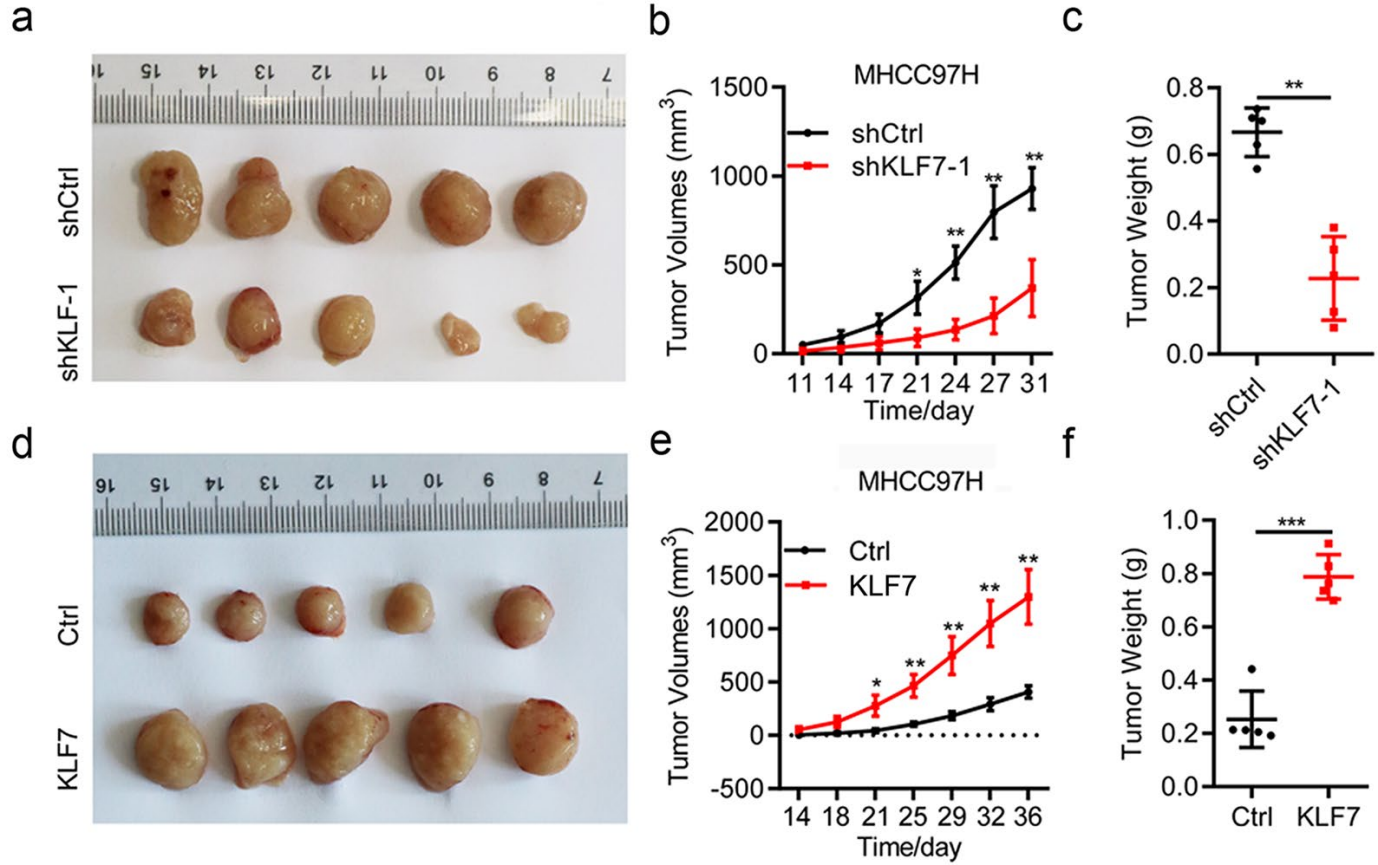
KLF7 expression affect tumor growth of HCC cells in vivo. **a** MHCC97H cells stably transfected with shKLF7 or shNC were subcutaneously injected into nude mice (n = 5). Four weeks later, mice were killed and photographed. **b** Tumor volume curves were drawn according to the tumor diameter. **c** Tumor weight of KLF7-knockdown nude mice was measured. **d** MHCC97H cells stably transfected with KLF7-overexpressing or empty lentivirus were subcutaneously injected into nude mice (n = 5). Representative images of tumors that striped from the nude mice four weeks later. **e** Tumor growth curve for the xenograft tumors. **f** Tumor weight of KLF7- overexpressing MHCC97H cells was presented. **p < 0.01, ***P < 0.001

To further investigate whether ectopic expression of KLF7 affects the growth of HCC xenograft tumors in vivo, a xenograft tumor model was established by subcutaneously injected shNC, sh-KLF7, empty vector or KLF7-overexpressing MHCC97H cells into the front flank of nude mice. As Fig. 4 suggested, the xenograft tumors transfected with sh-KLF7 grew much slower and with smaller tumor volume and weight than those transfected with shNC-MHCC97H cells (Fig. 4a–c, p < 0.01 for tumor weight). Correspondingly, the tumor size and weight were larger in mice injected with KLF7-overexpressing MHCC97H cells than tumors injected with empty vector cells (Fig. 4d–f, p < 0.001 for tumor weight). These results revealed that KLF7 expression affected tumor growth in vivo, which was consistent within in vitro study.

### KLF7 contributes to tumor progression by regulating VPS35 expression

**Fig. 5:**
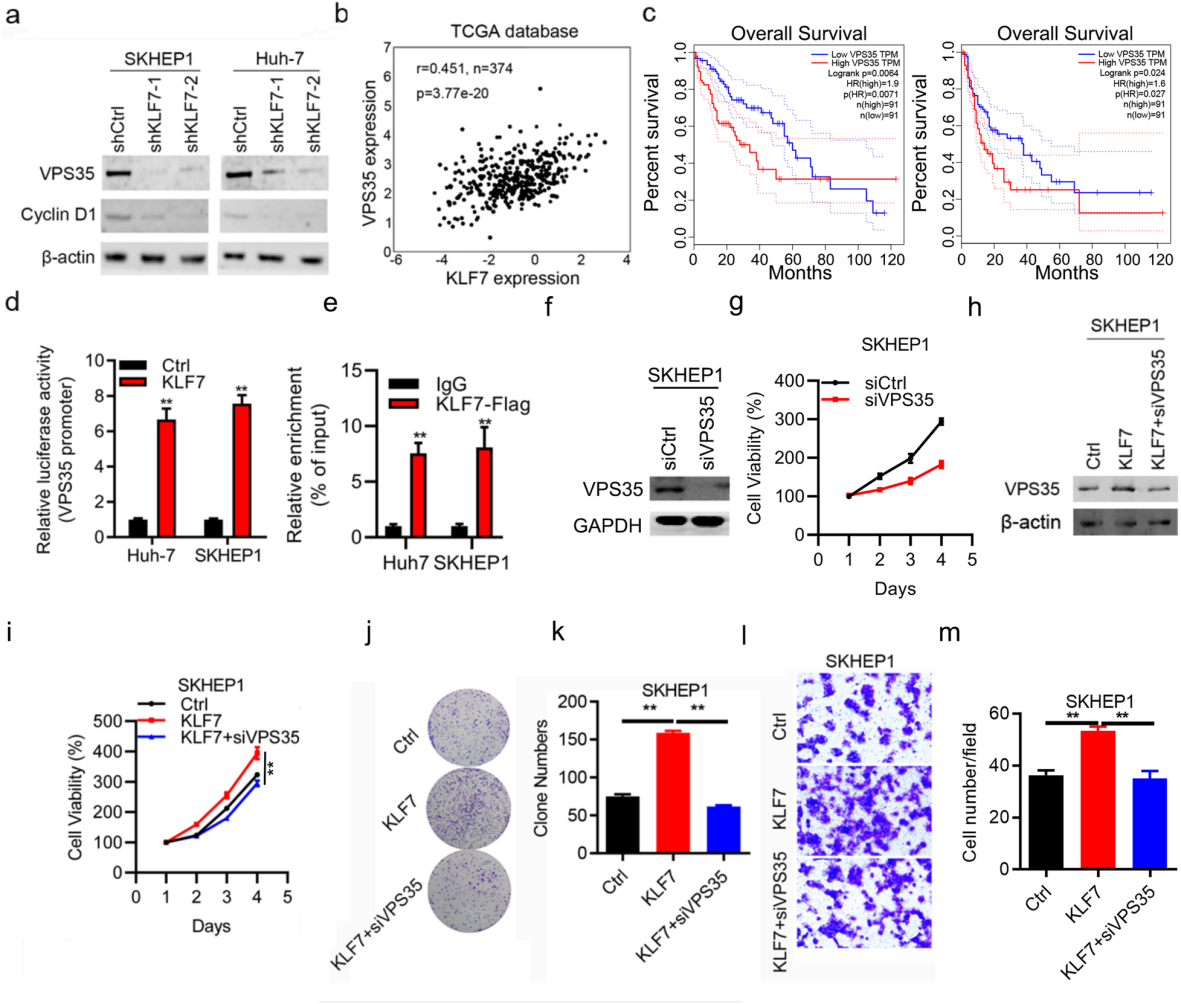
KLF7 contributes to tumor progression by regulating VPS35 expression. **a** Western blot analysis of VPS35 and cyclin D1 in si-KLF7 and siCtrl HCC cells. **b** A relationship between VPS35 and KLF7 was identified by TCGA database. **d** Luciferase reporter assay was used to determine the luciferase activity in HCC cells transfected with empty plasmids or plasmids containing VPS35. **e** ChIP-qPCR analysis of the interaction between KLF7 and VPS35 promoter sequence. **f** Western blot analysis of VPS35 in VPS35 knockdown HCC cells. **g** Cell viability of VPS35 knockdown HCC cells determined by CCK-8 assay. **h** Western blot analysis of VPS35 in KLF7 overexpression and VPS35 knockdown HCC cells. **i** Cell viability of KLF7 overexpression and VPS35 knockdown HCC cells determined by CCK-8 assay. **j**, **k** Representative images and statistical data of colony formation assay after simultaneously transfected with KLF7-overexpressing and VPS35-silencing SKHEP1 cells. **l**, **m** Representative images and statistical data of cell invasion assay after simultaneously transfected with KLF7-overexpressing and VPS35-silencing SKHEP1 cells. **p < 0.01

KLF7 functions as a critical transcription factor involved in the regulating of target gene expression in multiple biological events. We hypothesized that KLF7 medicated its effects on HCC tumor growth and invasion by regulating target gene expression. Western blots showed that knockdown of KLF7 suppressed VPS35 in SHEP1 and Huh-7 cell lines (Fig. 5a). Based on TCGA database analysis, there was a strong positive correlation between KLF7 expression and VPS35 expression (Fig. 5b). In addition, VPS35 high expression conferred poor overall and disease-free survival of HCC patients (Fig. 5c). To confirm the activity of the binding sites, we performed luciferase reporter assay and found that the reporter activity was increased by overexpression of KLF7 (Fig. 5d), which revealed that KLF7 has a transcriptional activation effect on VPS35 expression. CHIP-qPCR assay suggested that KLF7 could interact with the promoter of VPS35 (Fig. 5e).

Next, we investigated the role of VPS35 by silencing VPS35 in HCC cells. The results showed that VPS35 downregulation suppressed the proliferation of SKHEP1 cells (Fig. 5f, g). To further confirm that the observed phenotypes were medicated by the dysregulation of VPS35/KLF7 axis, several functional rescue assays were conducted. We found that upregulation of KLF7 promoted cell viability and colony formation were rescued by VPS35 knockdown, respectively (Fig. 5h–k, p < 0.01). In addition, silencing of VPS35 partially reverted the promotion of HCC cell invasion induced by the upregulation of KLF7 (Fig. 5l, m, p < 0.01). Collectively, our data demonstrated that KLF7 promotes HCC tumor growth and invasion by stimulating the expression of VPS35.

### *KLF7/VPS35 axis exerts its role on the HCC progression *via* enhancing the β-catenin signaling*

**Fig. 6:**
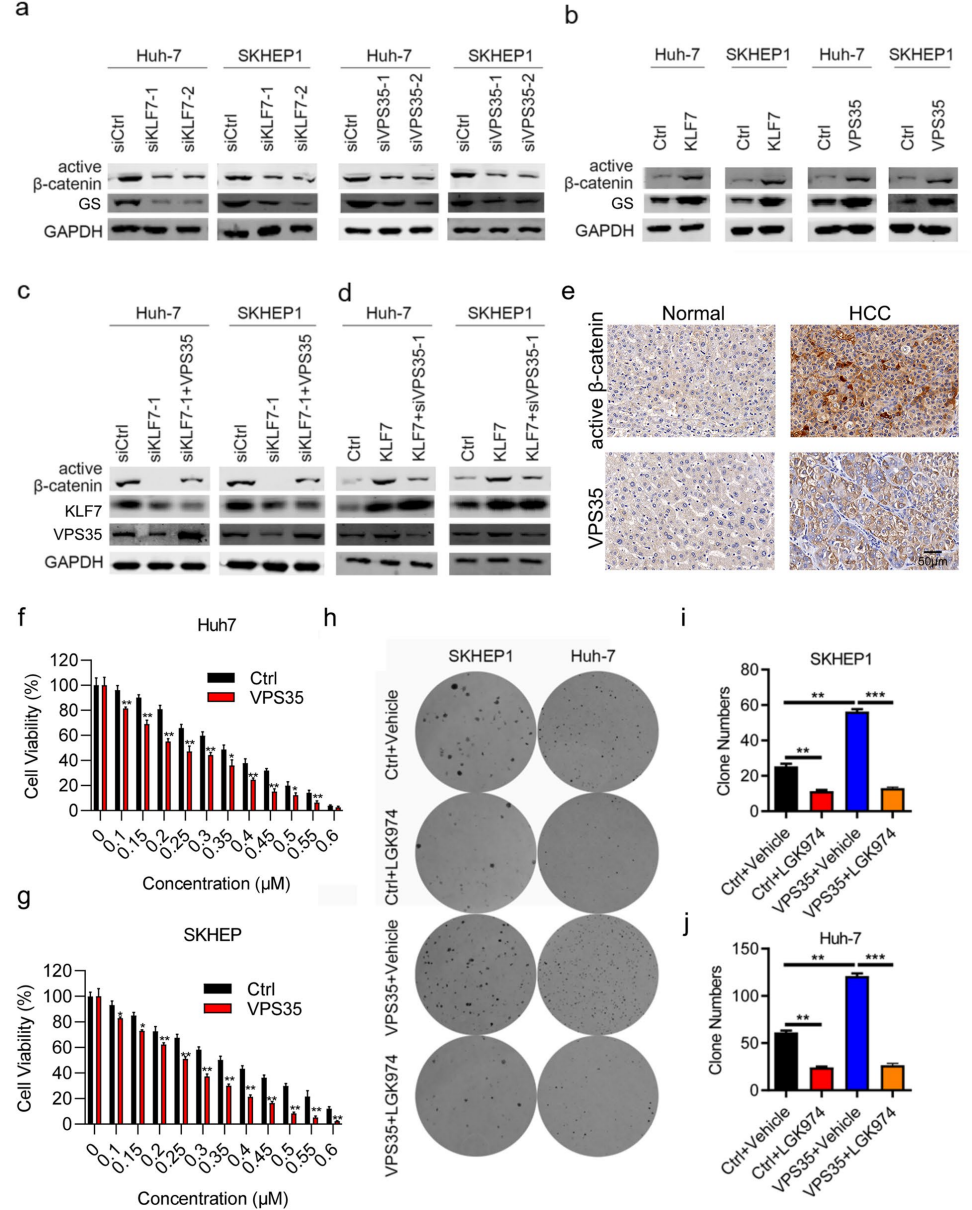
KLF7/VPS35 axis contributed to HCC progression via enhancing the β-catenin signaling. **a** Knockdown of KLF7 and VPS35 affected active-β-catenin and GS expression in HCC cells detected by western blot, respectively. **b** Overexpression of KLF7 and VPS35 affected active-β-catenin expression in HCC cells detected by western blot, respectively. **c** KLF7-silencing and VPS35-overexpressing affected active-β-catenin expression in Huh-7 and SKHEP1 cells. **d** KLF7-overexpressing and VPS35-silencing affected active-β-catenin level in Huh-7 and SKHEP1 cells. **e** The correlation between VPS35 and β-catenin expression in HCC and normal tissues. **f**, **g** Cell viability of VPS35-overexpressing HCC cells treated with GK974 in Huh-7 and SKHEP1 cells. **h** Representative images of colony formation assay in VPS35-overexpressing HCC cells treated with GK974. **i**, **j** Colony formation of VPS35-overexpressing cells treated with GK974 in Huh-7 and SKHEP1 cells

Previous studies have revealed that the β-catenin signaling pathway plays a crucial role in cancer cell progression. Therefore, we examined whether KLF7/VPS35 axis medicates the canonical β-catenin signaling. As shown in Fig. 6a, compared with control cells, the protein level of active-β-catenin, as well as its downstream indicator GS, was reduced in KLF7-silencing Huh-7 and SKHEP1 cells. Conversely, KLF7 overexpression had opposite effect (Fig. 6b). Additionally, downregulation of VPS35 reduced the expression of active-β-catenin, while VPS35 overexpression increased active-β-catenin expression in Huh-7 and SKHEP1 cells (Fig. 6a, b). Consistent results were also observed on the expression of GS (Fig. 6a, b). Subsequently, we found that β-catenin downregulation medicated by si-KLF7, which could be rescued by VPS35 overexpression in Huh-7 and SKHEP1 cells (Fig. 6c). Opposite results were obtained in HCC cells transfected with KLF7 overexpression and VPS35 knockdown (Fig. 6d). These data suggested that KLF7/VPS35 axis regulated β-catenin expression. Next, the clinical relation between expression of VPS35 and active-β-catenin was detected by IHC staining in HCC tissues. As expected, IHC staining results showed that both active-β-catenin expression was higher in cancer tissues comparing with normal tissues (Fig. 6e), which revealed a strong positive correlation between expression of VPS35 and active-β-catenin in HCC samples (Table 3). Thus, we examined cell survival of HCC cells transfected with VPS35-overexpressing or empty vectors, following treatment with β-catenin inhibitor, GK974. As Fig. 6f and g suggested, upregulation of VPS35 effectively improved chemosensitivity to β-catenin inhibitor, GK974, in HCC cells. While VPS35 overexpression increased cell colony formation, GK974 significantly suppressed colony formation ability in Huh-7 and SKHEP1 cells (Fig. 6h–j, all p < 0.001). In collusion, KLF7/VPS35 axis contributed to HCC cell growth via activating β-catenin signaling.

**Table 3:** Spearman correlation analysis of expression among KLF7, active β-catenin, and VPS35 in 50 HCC tissues by IHC

	KLF7		active β-catenin	
	*r*_*s*_	*P* value	*r*_*s*_	*P* value
Active β-catenin	0.633	< 0.001		
VPS35	0.456	< 0.01	0.475	< 0.01

r, Spearman correlation

### VPS35 interacts with Ccdc85c to participate in HCC progression

**Fig. 7:**
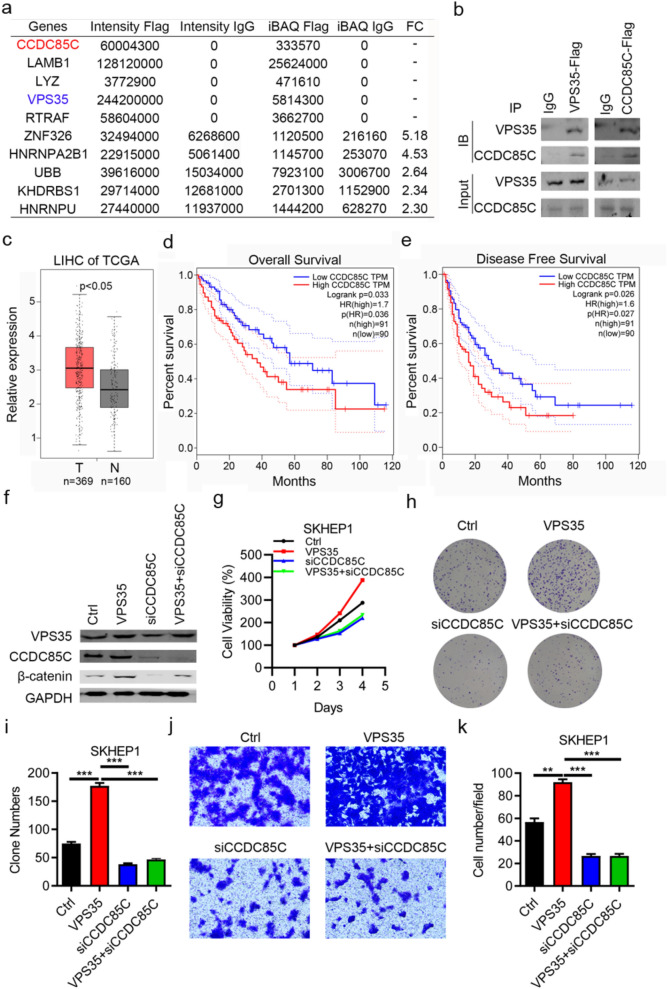
VPS35 interacts with Ccdc85c to participate in HCC progression. **a** Mass spectrometry analysis of VPS35-interacted proteins after co-IP. **b** Western blot was performed to confirm co-IP results. **c** The expression level of Ccdc85c in HCC patients and normal tissues by analyzed TCGA data. **d** The overall survival of HCC patients using KM-plotter by TCGA database analysis. **e** The disease-free survival of HCC patients predicted using KM-plotter. **f** Western blot determined Ccdc85c expression following siRNA or VPS35 overexpression. **g** CCK-8 assay was performed to examine cell viability following alteration of Ccdc85c and VPS35 expression in HCC cells. **h** colony formation and statistical data was performed to examine cell proliferation following alteration of Ccdc85c and VPS35 expression in HCC cells. **i** Statistical analysis of colony formation assay results. **j** Transwell assay was performed to examine cell invasion following alteration of Ccdc85c and VPS35 expression in HCC cells. **k** Statistical analysis of transwell assay results. **p < 0.01, ***P < 0.001

Given that KLF7 regulated VPS35 expression in HCC cells, we subsequently explored the underlying mechanisms of KLF7/VPS35 axis contributed to HCC progression by co-IP assay. As Fig. 6 revealed, VPS35 could interact with Ccdc85c in VPS35-overexpressing HCC cell lines, which was confirmed by mass spectrometry and western blot analysis (Fig. 7a, b). Additionally, the clinical relevance between expression of Ccdc85c and HCC patients suggested that Ccdc85c was high expressed in HCC patients, which was strongly associated with poor overall survival (p = 0.036) or disease-free survival (p < 0.027) of HCC patients (Fig. 7c–e). Further, rescue biological experiments, including cell viability, colony formation, transwell assay and cell apoptosis assay, were performed to investigate whether VPS35 regulated HCC progression via Ccdc85c. As western blot results suggested, Ccdc85c was obviously reduced by siRNA, which was upregulated by VPS35 overexpression (Fig. 7f). CCK-8 and colony formation assay showed that VPS35-overexpressing medicated cell proliferation was partly reverted by Ccdc85c knockdown in HCC cells (Fig. 7g–i), whereas overexpression of Ccdc85c effectively enhanced cell growth of HCC. By transwell assay, downregulation of Ccdc85c would rescue the invasion ability affected by VPS35 upregulation (Fig. 7j–k). These data clarified that VPS35 interacted with Ccdc85c to participate in HCC progression.

**Fig. 8:**
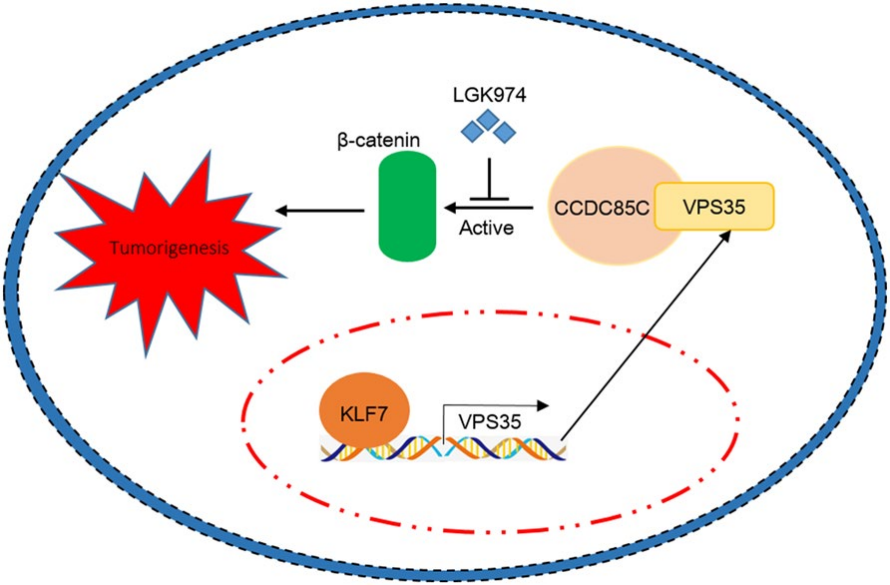
KLF7/VPS35 axis promoted hepatocellular carcinoma progression through CCDC85C-activated β-catenin pathway**.** A schematic graph of how KLF7/VPS35/CCDC85C/β-catenin axis promoted HCC growth

In summary, KLF7/VPS35 axis contributes to hepatocellular carcinoma progression through CCDC85C-mediated β-catenin pathway (Fig. 8).

## Discussion

Previous studies have reported that KLF family members are widely involved in the regulation of multiple cell processes, including cell proliferation, cell differentiation, cell apoptosis as well as cell cycle. Among them, KLF7 served as a transcription factor, which is required for neuronal morphogenesis [19]. Previous studies suggested that dysregulation of KLF7 played crucial roles in several solid tumors, including non-small-cell lung cancer and gastric cancer [11]. However, there is limited evidence mentioned the function of KLF7 in HCC. A deeper understanding of the functional landscape of KLF7 in the development of HCC is important for exploring novel and effective treatments. In the present study, we demonstrated that transcription factor KLF7 was overexpressed in human HCC samples. KLF7 overexpression also correlated with the metastasis status and low differentiation of the patients. Furthermore, KLF7 not only enhanced cell viability, invasion, cell cycle progression and blocked cell apoptosis in vitro, but also significantly augmented the tumorigenic capacity in vivo. In addition, KLF7 enhanced VPS35 transcription that promoted HCC tumor growth and invasion by activating beta catenin signaling pathway. Finally, we confirmed that β-catenin is essential for KLF7/VPS35 medicated cell proliferation, invasion and chemosensitivity using β-catenin inhibitor. Therefore, KLF7 functions as an oncogene in HCC.

As a member of KLF family, KLF7 plays an important role in the developing and adult nervous system [9]. Another study suggested that KLF7 was required for regulating TrkA gene expression and development of nociceptive sensory neurons [20]. Subsequently, studies suggested that overexpression of KLF7 regulated cell growth and progression by mediating STAT3-induced lncRNA LINC00668 in non-small-cell lung cancer, and contributed to squamous carcinoma progression [11, 21]. These above studies did not establish a direct player of KLF7 in promoting tumor growth and progression. Thus, in our study, we found that silence of KLF7 resulted in attenuated HCC tumor growth both in cultured cell and in mice study. These consistent findings suggest that KLF7 is an oncogene among different tumors. As a transcription factor, KLF7 has been shown to regulate a large number of target genes by regulating their expression. Therefore, it is reasonable that one or more target genes might be directly affected by KLF7 in HCC cells. According to the CHIP-seq and luciferase reporter results, we identified that VPS35 acted as a target gene of KLF7. In addition, we demonstrated a strong positive correlation between KLF7 and VPS35.

VPS35, a component of the retromer, was reported to be associated with sorting proteins to the trans-Golgi network or plasma membrane [22]. Recently, growing evidences have shown that VPS knockdown suppresses the proliferation of melanoma cells via inactivating the MAPK signaling [23], and results in cell apoptosis by disturbing the localization of Bcl-xL to mitochondrial membrane [24]. VPS35 is also upregulated in human HCC samples and contributes to HCC development [14]. However, the upstream regulator of VPS35 in HCC is unclear. Since KLF7 represents as important transcription factor which promotes the expression of target genes, whether KLF7 regulates VPS35 should be determined. Our study confirmed that overexpression of VPS35, regulated by KLF7, promoted the tumor growth in vitro and in vivo, and contributed to cell invasion, cell cycle and blocked cell apoptosis. These results were consistent with a recent published study, that demonstrated that VPS35 promoted the proliferation of hepatoma cell via the PI3K/AKT pathway [14].

Besides, the underlying mechanisms of KLF7/VPS35 axis medicated modulation of downstream pathways are not fully characterized. To hunt for some clues, we have performed co-IP assay to explore the downstream pathway of KLF7/VPS35 axis, which found that VPS35 could interacted with Ccdc85c in HCC cell lines. Ccdc85c, encoding a protein at apical junctions of radial glia, which may be involved in cell–cell adhesion and epithelium through interacted with β-catenin family [25]. The canonical β-catenin pathway plays important roles in cancer cell proliferation, invasion, apoptosis and metastasis. Our findings conformed that KLF7/VPS35 axis observably activated β-catenin signaling through interacting with Ccdc85c, to promote cell proliferation, colony formation, invasion and cell cycle of HCC cells, which was verified by detection active β-catenin expression following alteration of KLF7 and VPS35 expression, and biological functions studies treated with β-catenin inhibitor in HCC cell lines. In addition, a strong positive correlation between VPS35 and β-catenin was observed in HCC tissues. Collectively, these results revealed that KLF7/VPS35 axis promoted HCC cell proliferation, invasion and cell cycle, as well as blocked cell apoptosis by activating Ccdc85c-medicated β-catenin pathway.

There were some limitations in the present study. For example, the role of KLF7 in HCC cell metastasis in vivo was not examined. The association of KLF7, VPS35 and β-catenin with HCC patients’ prognosis was not analyzed from our patients. In the future, we will collect the patients’ survival information to finish this analysis. We will also investigate the significance of KLF7 on tumor metastasis in vivo.

In summary, our study demonstrated the crucial roles of KLF7/VPS35 axis in HCC tumor growth, cell invasion, cell cycle and cell apoptosis. We revealed that KLF7 regulated VPS35 expression by acting as a transcription factor in HCC cells. In addition, KLF7 promoted HCC cell proliferation, invasion, cell cycle and blocked cell apoptosis by enhancing Ccdc85c-medicated β-catenin pathway, which might be a worthwhile therapeutic target for HCC.

## Data Availability

The datasets used and/or analyzed during the current study are available from the corresponding author on reasonable request.
